# Dissecting the cryoprotection mechanisms for dehydrins

**DOI:** 10.3389/fpls.2014.00583

**Published:** 2014-10-29

**Authors:** Cesar L. Cuevas-Velazquez, David F. Rendón-Luna, Alejandra A. Covarrubias

**Affiliations:** Departamento de Biología Molecular de Plantas, Instituto de Biotecnología, Universidad Nacional Autónoma de MéxicoCuernavaca, México

**Keywords:** dehydrins, late embryogenesis abundant proteins, cryoprotection, water deficit, abiotic stress, intrinsically disordered proteins

## Abstract

One of the common responses of plants to water deficit is the accumulation of the so-called late embryogenesis abundant (LEA) proteins. *In vitro* studies suggest that these proteins can protect other macromolecules and cellular structural components from the impairments caused by water limitation. Their binding to phospholipids, nucleic acids and/or to divalent cations has suggested multi-functionality. Genetic analyses indicate that these proteins are required for an optimal adjustment of plants to this insult. This diverse information has conducted to propose different models for LEA proteins action mechanisms. Many of these properties are shared by group 2 LEA proteins or dehydrins (DHNs), one of the LEA protein families for which large amount of data is available. This manuscript focuses on the different mechanisms proposed for this LEA protein group by analyzing published data derived from *in vitro* cryoprotection assays. We compared the molar ratio of protectant:enzyme needed to preserve 50% of the initial activity per enzyme monomer to assess different mechanisms of action. Our results add evidence for protein–protein interaction as a protection mechanism but also indicate that some DHNs might protect by different means. The strength and weakness of the proposed protection mechanisms are discussed.

## DEHYDRINS, A PLANT SPECIFIC GROUP OF LEA PROTEINS

Late embryogenesis abundant (LEA) proteins are a group of enigmatic proteins that have been strongly associated with plant responses to water deficit (Battaglia et al., 2008; Hincha and Thalhammer, 2012). They accumulate mainly in dry seeds but also in vegetative tissues when plants experience water deficit such as drought, freezing and high salinity. Among the different LEA proteins, group 2 LEA proteins (D11) have been the most studied (Rorat, 2006; Hara, 2010; Hanin et al., 2011). Proteins in this group are also known as dehydrins (DHNs), and to date they have been found only in plants (Battaglia et al., 2008; Hara, 2010). Due to the vast majority of DHNs reports over other LEA proteins, people not familiar with these proteins assume that all LEA proteins are DHNs; however, this is not the case, LEA proteins represent a rather large group of diverse proteins. Depending on sequence similarity and the presence of particular motifs, LEA proteins have been classified in at least seven groups or families (Battaglia et al., 2008). Although there is sequence similarity within proteins in each group, different LEA protein groups exhibit virtually no sequence conservation with each other. Nevertheless, typical LEA proteins share distinctive physicochemical characteristics such as high hydrophilicity, high content of Gly, Ala and Ser, and lack or underrepresentation of Cys, Trp, and other hydrophobic amino acids (Dure, 1993; Garay-Arroyo et al., 2000; Hundertmark and Hincha, 2008). These characteristics suggest that LEA proteins from different groups might have similar functional properties.

Computational analyses have indicated that, as other LEA proteins, DHNs lack stable tridimensional structures, leading to be considered as intrinsically disordered proteins (IDPs; Close, 1996; Battaglia et al., 2008). Some of them have been experimentally characterized as IDPs in solution (Ismail et al., 1999; Soulages et al., 2003; Bokor et al., 2005). In spite of this structural flexibility, it has been shown that some DHNs gain ordered structure in the presence and binding to other molecules such as lipids, nucleic acids, or metal ions (Koag et al., 2003; Hara et al., 2009; Eriksson et al., 2011; Rahman et al., 2011).

Dehydrins have been defined by the presence of a Lys-rich segment, or K-segment, which may be repeated several times. Moreover, they are further classified in sub-classes depending on the representation of some of their distinctive conserved motifs, such as the Y-segment (Tyr-rich), or the S-segment (Ser-track). Accordingly, five sub-classes can be distinguished: K_(n)_, SK_(n)_ K_(n)_S, Y_(n)_K_(n)_, and Y_(n)_SK_(n)_ (n = number of repeats), for each of which different functions have been proposed (Hara, 2010; Hanin et al., 2011).

Several approaches have been followed to determine the function of these proteins, using *in vivo* and *in vitro* experimental systems. Even though for some LEA protein groups it has been shown their participation in the response of vascular plants to water limiting environments by reverse genetics (Manfre et al., 2006; Kim and Nam, 2010; Olvera-Carrillo et al., 2010), this has not been the case for DHNs, mostly due to the large number of members found within this family (*Arabidopsis* group 2 LEA proteins presents 10 members; Battaglia et al., 2008; Hundertmark and Hincha, 2008). However, a contribution to salt and osmotic stress tolerance was reported for the two *DHN* genes (*PpDHNA* and *PpDHNB*) of the moss *Physcomitrella patens*, for which targeted knockout mutants were characterized (Saavedra et al., 2006; Ruibal et al., 2012). Binding to other macromolecules such as negatively charged lipids and DNA (Koag et al., 2003; Hara et al., 2009) have led to propose that DHNs can protect the integrity of biological membranes and nucleic acids from the effects caused by low water availability. Also, the binding to divalent cations such as Ca^2+^, Zn^2+^, Fe^3+^, Co^2+^, Ni^2+^, and Cu^2+^ has suggested that DHNs might act as buffer for these metals under water deficit (Kruger et al., 2002; Alsheikh et al., 2005; Hara et al., 2005). The ability of DHNs to bind to such diverse set of ligands could be due to their structural flexibility.

An extensively explored possibility for DHNs function has been their competence to protect other proteins from the effects resulting from water scarcity in the cellular environment. This hypothesis has been addressed by *in vitro* assays, where water limitation is imposed by partial water dehydration or by freeze/thaw cycles. The results from these experiments have demonstrated that different DHNs can prevent the inactivation of reporter enzymes [lactate dehydrogenase (LDH); alcohol dehydrogenase (ADH); firefly luciferase; citrate synthase (CS); β-glucosidase G, (βglG); and glucose oxidase (GOD/POD)] under these different water deficit conditions indicating that some of them are cryo- and/or dehydro-protectors (Sanchez-Ballesta et al., 2004; Tantos et al., 2009; Brini et al., 2010; Drira et al., 2013).

## DISSECTING THE MOLECULAR MECHANISM OF DHNs PROTEIN PROTECTION

Several years ago, it was reported that addition of some LEA proteins, including DHNs, prevented the inactivation of reporter enzymes upon various freezing and thawing cycles (Lin and Thomashow, 1992; Kazuoka and Oeda, 1994). This *in vitro* assay is now a common approach to evaluate the protective activity of LEA proteins under these stress condition (Wisniewski et al., 1999; Hara et al., 2001; Bravo et al., 2003; Momma et al., 2003; Sanchez-Ballesta et al., 2004; Reyes et al., 2008; Hughes and Graether, 2011; Hughes et al., 2013). Kazuoka and Oeda (1994) reported that SoCOR85, an 85 KDa DHN from spinach, was able to keep LDH activity after freezing the sample for 24 h at -20∘C and subsequent thawing at 4∘C for 2 h. In that work, SoCOR85 showed a PD_50_ (“Protectant protein” concentration needed to preserve 50% of the reporter enzyme activity) smaller than that for a variety of proteins with no relation to DHNs, including BSA, a known cryoprotectant. These data indicated that SoCOR85 was an effective cryoprotectant, and suggested that this cryoprotective activity was specific (Kazuoka and Oeda, 1994). Subsequently, a similar activity was found for DHNs from different plant species such as TaWCS120 from wheat (Houde et al., 1995), PpPCA60 from peach (Wisniewski et al., 1999), CuCOR19 from *Citrus unshiu* (Hara et al., 2001), HvP-80/Dhn5 from barley (Bravo et al., 2003), GmDHN26 and GmDHN27 from soybean (Momma et al., 2003), CrCOR15 from fortune mandarin fruit (Sanchez-Ballesta et al., 2004), ERD10 from *Arabidopsis* (Reyes et al., 2008), RcDhn5 from *Rhododendron catawbiense* Michaux (Reyes et al., 2008), ERD14 from *Arabidopsis* (Tantos et al., 2009), TaDHN-5 from wheat (Drira et al., 2013), VrYSK_2_ from *Vitis riparia* (Hughes and Graether, 2011; Hughes et al., 2013), TsDHN-2 from *Thellungiella salsuginea* (Hughes et al., 2013), and OpsDHN-1 from *Opuntia streptacantha* (Hughes et al., 2013).

**FIGURE 1:**
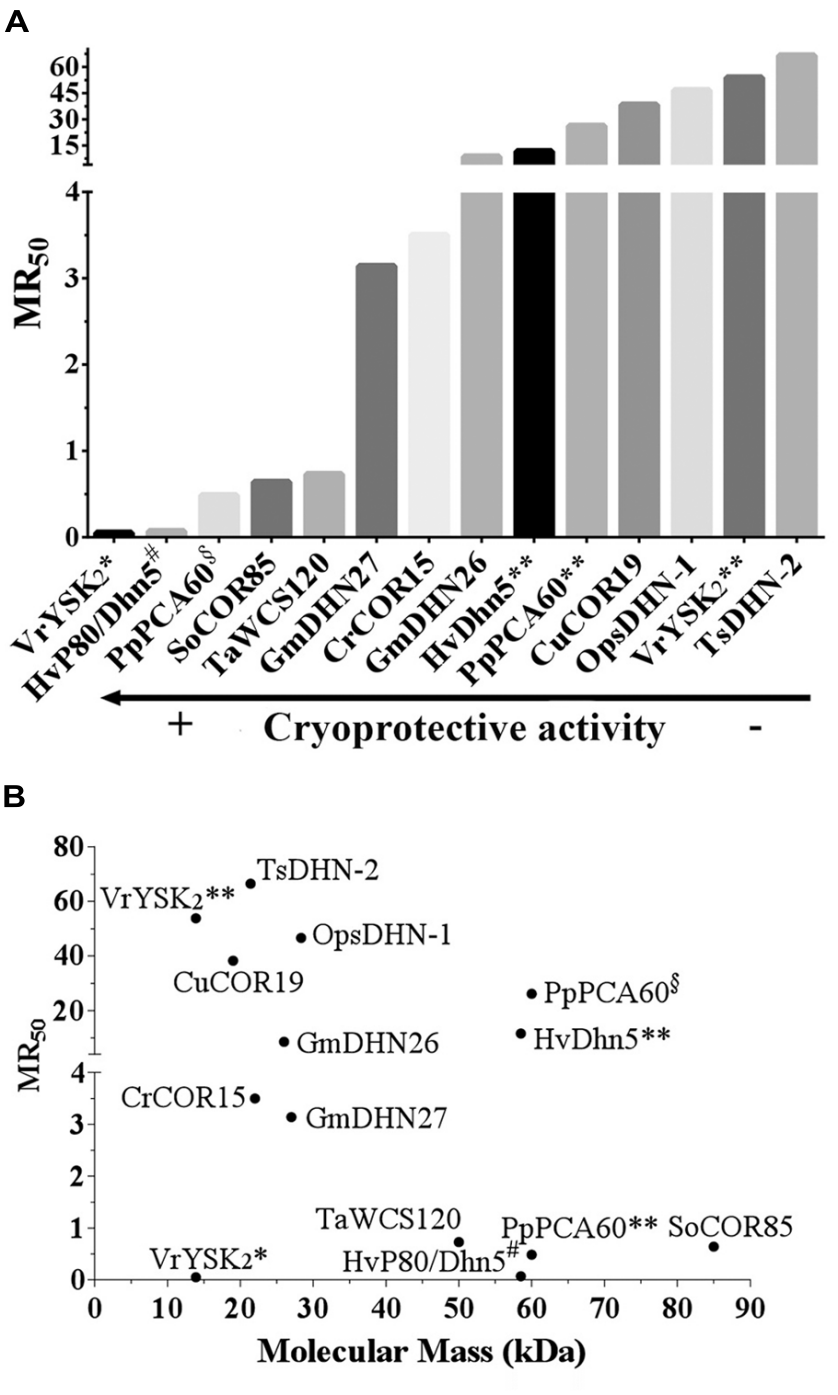
**(A)** Cryoprotective efficiency of DHNs. The DHN considered for this analysis were those for which the available data allowed to calculate the amount of molecules needed to preserve 50% of the initial activity per molecule of the reporter enzyme or molecular ratio 50 (MR_50_). The data used for these calculations and the resulting MR_50_ values are shown in Supplementary Table 1. **(B)** MR_50_ values for DHNs with different molecular mass. A conflict in the reported data leading to contrasting cryoprotective efficiency was found in the following cases: VrYSK2* (Hughes and Graether, 2011), VrYSK2** (Hughes et al., 2013), PpPCA60^§^ (Wisniewski et al., 1999), PpPCA60** (Hughes et al., 2013), HvDhn5** (Hughes et al., 2013), and HvP80/Dhn5^#^ (Bravo et al., 2003). MR_50_ values were calculated using the following equation: MR_50_ = {(36.4)*PD_50_}/{[LDH]*MM}, where 36.4 is the molecular mass of LDH monomer in kDa; PD_50_ in μg/mL; [LDH]: LDH concentration in μg/mL; MM: Dehydrin’s molecular mass.

Although there are some differences in the *in vitro* assays cited above (Supplemental Table 1), the data from this experimental system seem to be a good platform to compare the effectiveness of this activity among the different DHNs tested, and to address questions regarding the mechanism of action involved in such protecting effect. In order to be able to compare the data from these different reports, we have unified this information by considering not only the amount but also the molecular mass of the proteins assayed; hence, instead of comparing PD_50_ we compared the molar ratio of protectant:enzyme, which ponders the amount of molecules needed to preserve 50% of the initial activity per monomer of the reporter enzyme (Molar Ratio_50_, MR_50_). LDH was the common reporter enzyme in all cases analyzed here. The original and standardized data are shown in Supplemental Table 1. The comparison between the MR_50_ of the different DHNs showed a broad range of cryoprotection levels, from 0.05 to 66.5. Hv80/Dhn5 showed to be the most effective cryoprotectant with a MR_50_ of 0.07 indicating that only one molecule (or less) of DHN is required to protect one molecule of LDH monomer during freeze/thaw treatments (**Figure 1A**). Even considering TsDHN2, the less effective protectant with a MR_50_ of 66.5, DHNs cryoprotection effectiveness seems remarkable when it is compared with that attained by compatible osmolytes such as sucrose, for which 10^6^ molecules are needed to protect one monomer of LDH (Houde et al., 1995).

**FIGURE 2:**
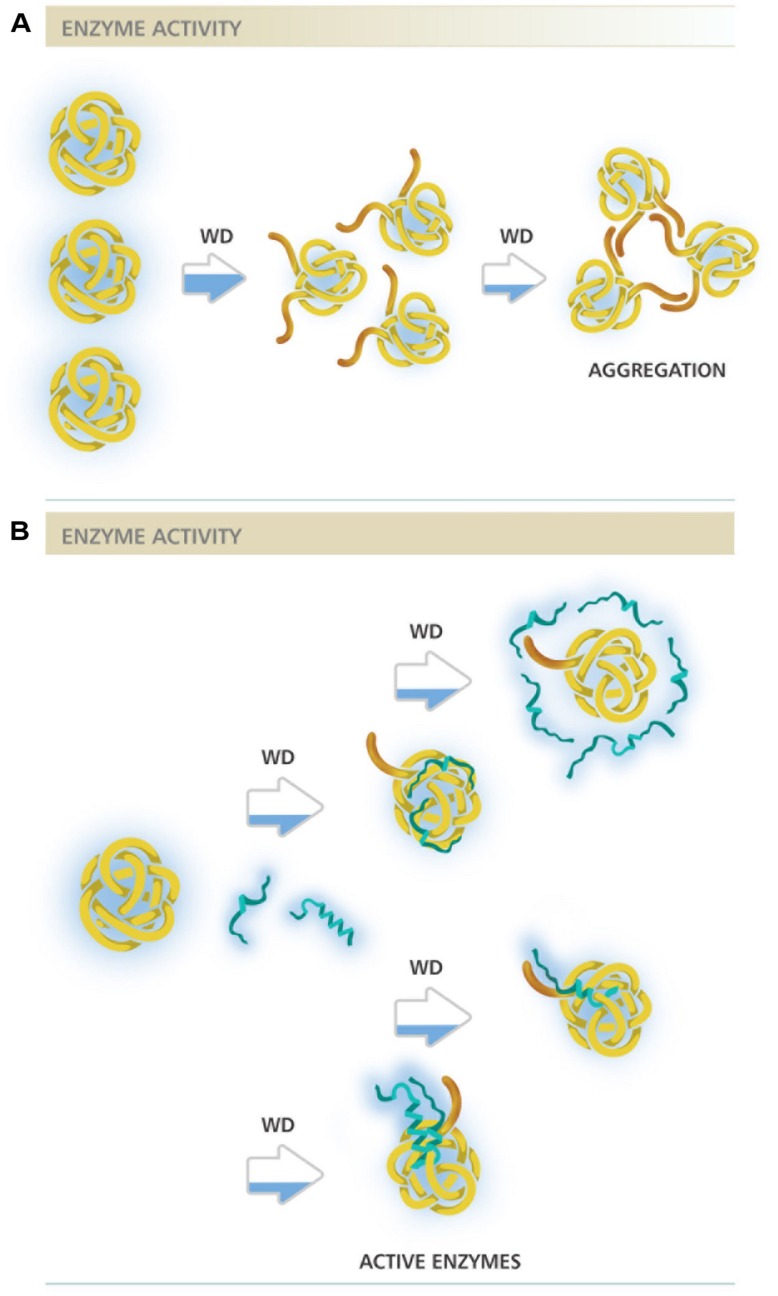
**Different molecular mechanisms proposed for DHNs protective activity. (A)** Representation of an enzyme under water deficit in the absence of DHNs. Brown extensions represent enzyme hydrophobic regions starting to be exposed as a consequence of low water availability, indicated by the partially blue filled arrows. A severe dehydration may lead to a higher exposure of hydrophobic regions resulting in enzyme aggregation. **(B)** Representation of an enzyme under water deficit in the presence of DHNs. Blue strands represent DHN molecules in two possible structural conformations, with high structural disorder and with some helicity possibly gained under water limitation. The different mechanisms proposed are represented after each partially blue filled arrow. From top to bottom: the first two figures illustrate protection by molecular shield, where DHN molecules not necessarily interact with their targets but rather their proximity maintain the amount and organization of water molecules needed to keep enzyme integrity. A variant of this mechanism considers their hydrodynamic radius, a highly disordered DHN molecule could present a large hydrodynamic radius able to cover a larger surface of its target; the two bottom figures represent two variants of the mechanism where protein–protein interaction is required to select and protect their targets, in one case DHN can bind as monomers whereas in other as dimer or any other oligomeric form. The filling in the horizontal bars above schemes represents enzyme activity level.

This analysis allows envisaging at least two different molecular mechanisms of action to explain DHNs protective activity, both already proposed in different reports (Reyes et al., 2005; Tunnacliffe and Wise, 2007; Tompa and Kovacs, 2010; Olvera-Carrillo et al., 2011; Chakrabortee et al., 2012; Hughes et al., 2013). One, supported by the low DHN:enzyme molar ratios needed to get protection, in which protein–protein interaction is strongly suggested, and another where a higher amount of DHN molecules seem to be necessary to exert this protective effect (**Figures 1A and 2**). The first hypothesis is also supported by additional published data that was not considered in this study because the available data did not allow estimating MR_50_ values. This is the case for ERD10, an *Arabidopsis* DHN, which was able to keep 75% of LDH activity after five freeze/thaw cycles in a 1:1 (ERD10:LDH) molar ratio, suggesting a MR_50_ lower than 1 (Reyes et al., 2008). Similarly, ERD14 from *Arabidopsis* preserved 100% of the ADH activity when subjected to five freeze/thaw cycles in a 0.5:1 (ERD14:LDH) molar ratio (Tantos et al., 2009).

Those cases with a high MR_50,_ where many DHN molecules are required for cryoprotection, suggest a mechanism where DHN molecules would not necessarily need to contact the cryo-susceptible target protein but rather stay localized near the enzyme and, because their hydrophilic and highly disordered characteristics, they may provide an appropriate environment to stabilize a native and functional structure, a mechanism that has been referred as molecular shield (Tunnacliffe and Wise, 2007; Chakrabortee et al., 2012; Hughes et al., 2013). For this last mechanism, it has been proposed that the extended random coil structure reported for some of the characterized DHNs would result in proteins with a large hydrodynamic radius, which predicts that DHNs with this conformation would be able to align their hydrophilic amino acids around the surface of a target protein to circumvent the loss of its bulk water and consequent changes in its native structure (Tunnacliffe and Wise, 2007; Hughes et al., 2013). This hypothesis is supported by a recent work, where different concatemers of the K_2_ hypothetical protein from *V. riparia* were used (Hughes et al., 2013). In this report it was found that the level of protection conferred by these K-segment concatemers was directly proportional to their hydrodynamic radius. A similar correlation was observed by comparing the protection level of polyethylene glycol (PEG) polymers with different hydrodynamic radius. By contrast, no correlation was detected when globular proteins were tested (Hughes et al., 2013). However, if we consider the MR_50_ for these proteins (Supplemental Table 1), they showed values between 39.3 and 668 (up to 668 molecules of DHN are needed to protect one molecule of LDH). From these data, it is evident that the cryoprotective efficiency of these concatemers is far low from that showed by other DHNs, where protection was detected even with one protein molecule (**Figure 1A**; Supplemental Table 1), suggesting that cryoprotection through molecular shield is less efficient than that obtained by protein–protein interaction. The need of a larger number of molecules that could be protecting through a molecular shield mechanism could also be interpreted as an unspecific effect produced just by the presence of many molecules close to a sensitive target, which also could occur by some other proteins apparently not devoted to such specific function. If only the hydrodynamic radius and the hydrophilicity of a protecting protein are relevant for this effect, then any polypeptide with these properties despite their amino acid sequence would be a cryoprotectant, which raises a question remaining to be addressed. On this regard, it has been reported that a large and highly hydrophilic amino acid-based polymer, poly L-Lysine was unable to protect LDH activity from freeze/thaw treatments (Reyes et al., 2008), implying the contribution of additional characteristics in a protein to be a good protectant. The relevance of the protein sequence also has to be considered for the data obtained using the K_2_ concatemers because the proteins obtained not only are longer with larger hydrodynamic radius but they also contain an increasing number of the conserved K-segments, whose specific sequence could influence on this effect. The participation of K-segments in DHNs cryoprotection was suggested because their progressive deletion from *Arabidopsis thaliana* and *Rhododendron catawbiense* DHNs (ERD10 and RcDhn5, respectively) impaired their ability to protect LDH activity (Reyes et al., 2008). Similar findings were obtained for wheat DHN-5 (Drira et al., 2013).

The MR_50_ from many of the DHN cryoprotection experiments reported also allowed us to look for a relation between the size of natural DHNs and their molecular protection effectiveness; however, no correlation was found between these two parameters (**Figure 1B**). There are low molecular mass DHNs with an MR_50_ close to 70 and others whose MR_50_ was lower than 10. Although some correlation could be detected for the K-segments analyzed by Hughes et al. (2013; see Supplementary Table 1), the data for any set of the natural DHNs, with low or high molecular mass (**Figure 1B**), are not consistent with a general mechanism in which the length of DHNs plays an important role in cryoprotection. Furthermore, we neither found any correlation by considering the levels of structural disorder determined using PONDR tools (Romero et al., 1997). HvP80/Dhn5 with the highest cryoprotection efficiency shows the same level of disorder (52.7%) than that obtained for TsDHN2 (52.43%) with the lowest cryoprotection efficiency, indicating that the protective effect is rather related to specific properties in each of the DHNs tested.

Results where direct binding between a reporter enzyme and a DHN was not detected favored the idea that the physicochemical properties of LEA proteins (including DHNs), such as the abundance of charged amino acid residues, promote electrostatic interactions to keep the two proteins closely enough to provide protection without binding (Hughes and Graether, 2011). However, data indicating that one or few LEA protein molecules are enough to protect reporter enzymes from the effects of water scarcity (Kazuoka and Oeda, 1994; Houde et al., 1995; Bravo et al., 2003; Reyes et al., 2005; Nakayama et al., 2007) sustains the possibility of direct protein interaction, which is further supported by the analysis made in the present work. Moreover, evidence for a physical interaction between LEA proteins and target proteins has been obtained for some hydrophilins and LEA proteins (Alsheikh et al., 2005; Reyes et al., 2005; Nakayama et al., 2007; Kushwaha et al., 2012, 2013; Xie et al., 2012). This has also been the case for Y_2_K_4_-type DHN from *Medicago truncatula*, for which it was found *in vitro* and *in vivo* interaction with an ICE-type (inducer of CBF expression 1) transcription factor (Xie et al., 2012). The low occurrence of hydrophobic patches in the many DHNs and other LEA proteins predicts low affinity association with other proteins; however, attention should be given to their amino acid sequence as well as to possible structural modifications that could be promoted by changes in their environment (Mouillon et al., 2008; Olvera-Carrillo et al., 2011) and/or by their interaction with their clients, as it has been proposed for various IDPs (Dyson and Wright, 2005; Tompa and Fuxreiter, 2008; Pancsa and Tompa, 2012). Based on the physicochemical properties of IDPs and some experimental evidence (Pufall et al., 2005; Galea et al., 2008; Wang et al., 2011), it has also been hypothesized that the existence of a variety of structural states for a particular IDP or intrinsically disordered regions (IDRs) could lead to the formation of dynamic protein complexes, where this macromolecular ensembles may fluctuate between diverse structural organizations (Tompa and Fuxreiter, 2008). Hence, protein–protein interactions between LEA protein and their clients may be transient, making more challenging the finding of experimental conditions where these interactions could be stabilized.

## FUTURE DIRECTIONS

Overall, considering the differences in the reported evidence and the analysis in this study, it cannot be discarded that different DHNs perform their protective function by different mechanisms or a combination of them (see **Figure 2**) depending on their particular sequences or even on the severity of the stress and/or cell type where they carry out their function. The possibility that DHNs perform multiple functions, a feature that seems to be common for IDPs (Jeffery, 2003; Sun et al., 2013), is something to be considered for their molecular mechanisms of action. Evidence for multi-functionality has been obtained *in vitro* for some DHNs, showing their ability to bind divalent cations, nucleic acids or some phospholipids, in addition to their cryoprotective effect (Koag et al., 2003, 2009; Hanin et al., 2011). It should be kept in mind the possible role of post-translational modifications in the modulation of different functions and interactions, considering a further structural and functional characterization of DHNs obtained from plant tissues. Their potential role in plant cells as protectant proteins or chaperones during water deficit needs additional evidence, identifying their natural targets or clients as well as regions or sequences in their structure relevant for their function.

The structural disordered nature of DHNs, their distinctive properties and sequences impose *ad hoc* experimental designs, some of them challenging but needed to get closer to the understanding of their function in the plant responses to abiotic and biotic stress. It is imperative the analysis of a larger number of DHNs as well as the standardization of *in vitro* protection assays to be able to address various aspects of the proposed mechanisms. The emergence of new and high-resolution technologies represents a good opportunity to validate the proposed molecular mechanisms and to address their relevance in the plant cell.

## Conflict of Interest Statement

The authors declare that the research was conducted in the absence of any commercial or financial relationships that could be construed as a potential conflict of interest.
