# Supplementary material for: Dissecting the cryoprotection mechanisms for dehydrins
Source: Front Plant Sci. 2014 Oct 29;5:583. doi: 10.3389/fpls.2014.00583 (PMC4212605; doi:10.3389/fpls.2014.00583)
Supplement: Supplementary file 1 [file Table_1.PDF]

Supplemental Table 1. Cryoprotective efficiencies of the different dehydrins cited in this study.

Dehydrin	MM (kDa)	Source	(Freeze/Thaw) Cycles [a]	[LDH] (µg/ml)	[LDH][b] (nM)	PD_{50} (µg/ml)	PD_{50} (nM)	MR_{50}	Reference
SoCOR85	85	*Spinacia oleracea*	(1°C min^{-1} until -20°C, 24 h/ 4°C, 1-2 h) 1x	10	275	15	176	0.64	Kazouka & Oeda (1994)
TaWCS120	50	*Triticum aestivum*	(1°C min^{-1} until -20°C, 24 h/ 4°C, 1-2 h) 1x	10	275	10	200	0.73	Houde *et al.* (1995)
PpPCA60	60	*Prunus persica*	(Liquid N_2, 30 sec/ RT, 5 min) 9x	25	687	20	333	0.485	Wisniewski *et al.* (1999)
CuCOR19	19	*Citrus unshiu*	(Liquid N_2, 1 min/ RT, 4 min) 1x	0.4	11	8	421	38.3	Hara *et al.* (2001)
HvP80/Dhn5	58.5	*Hordeum vulgare*	(Liquid N_2, 30 sec/ RT, 5 min) 9x	25	687	2.8	48	0.07	Bravo *et al.* (2003)
GmDHN26	26	*Glycine max cv. Miyagi-ao & cv. Tanbaguro*	(-20°C, 24h/ RT, 5-10 min) 1x	1.25	35	7.8	300	8.57	Momma *et al.* (2003)
GmDHN27	27	*Glycine mac cv. Nattosyoru*	(-20°C, 24h/ RT, 5-10 min) 1x	1.25	35	2.97	110	3.14	Momma *et al.* (2003)
CrCOR15	22	*C. clementina X C. reticulata*	(Liquid N_2, 30 sec/ RT, 5 min) 2x	9.1	250	19.25	875	3.5	Sánchez-Ballesta *et al.* (2004)
VrYSK$_2$	13.9	*Vitis riparia*	(Liquid N_2, 30 sec/ 4°C, 5 min) 5x	25	687	0.5	36	0.05	Hughes & Graether (2011)
K_2	5.4	*Vitis riparia*	(Liquid N_2, 30 sec/ 4°C, 5 min) 5x	25	687	1.1	204	0.3	Hughes & Graether (2011)
PpPCA60	60	*Prunus persica*	(Liquid N_2, 30 sec/ 4°C, 5 min) 5x	25	687	1080	18,000	26.2	Hughes *et al.* (2013)
HvDhn5	58.5	*Hordeum vulgare*	(Liquid N_2, 30 sec/ 4°C, 5 min) 5x	25	687	468	8,000	11.64	Hughes *et al.* (2013)
K_{10}	35.7	*Vitis riparia*	(Liquid N_2, 30 sec/ 4°C, 5 min) 5x	25	687	964	27,000	39.3	Hughes *et al.* (2013)
OpsDHN-1	28.36	*Opuntia streptacantha*	(Liquid N_2, 30 sec/ 4°C, 5 min) 5x	25	687	905.6	32,000	46.6	Hughes *et al.* (2013)
K_8	28.2	*Vitis riparia*	(Liquid N_2, 30 sec/ 4°C, 5 min) 5x	25	687	874.2	31,000	45.1	Hughes *et al.* (2013)
TsDHN-2	21.4	*Thellungiella salsuginea*	(Liquid N_2, 30 sec/ 4°C, 5 min) 5x	25	687	963	45,000	66.5	Hughes *et al.* (2013)
K_6	20.7	*Vitis riparia*	(Liquid N_2, 30 sec/ 4°C, 5 min) 5x	25	687	807.3	39,000	56.76	Hughes *et al.* (2013)
VrYSK$_2$	13.9	*Vitis riparia*	(Liquid N_2, 30 sec/ 4°C, 5 min) 5x	25	687	515	37,000	53.85	Hughes *et al.* (2013)
K_4	13.1	*Vitis riparia*	(Liquid N_2, 30 sec/ 4°C, 5 min) 5x	25	687	799	61,000	88.8	Hughes *et al.* (2013)
K_2	5.4	*Vitis riparia*	(Liquid N_2, 30 sec/ 4°C, 5 min) 5x	25	687	659	122,000	177.6	Hughes *et al.* (2013)
KK	3.2	*Vitis riparia*	(Liquid N_2, 30 sec/ 4°C, 5 min) 5x	25	687	806.4	252,000	366.8	Hughes *et al.* (2013)
K	1.7	*Vitis riparia*	(Liquid N_2, 30 sec/ 4°C, 5 min) 5x	25	687	780.3	459,000	668	Hughes *et al.* (2013)

Only the reports where MR_{50} could be calculated are shown. MR_{50} was calculated using the following equation: $MR_{50} = \{(36.4)*PD_{50}\}/\{[LDH]*MM\}$, where 36.4 is the molecular mass of LDH monomer in kDa; PD_{50} in µg/mL; [LDH]: LDH concentration in µg/mL; MM is the protectant molecular mass in kDa.

[a] Short description of the freeze/thaw treatment used in each case. First two lines describes a progressive freezing treatment with 1°C reduction down to -20°C, and thaw at 4°C. RT: room temperature; x: number of freeze/thaw cycles.

[b] LDH concentration was calculated relative to monomer (36 kDa).
